# New Obstetrical Journal

**Published:** 1848-01

**Authors:** 


### THE MEDICAL EXAMINER.

PHILADELPHIA, JANUARY, 1848.

### NEW OBSTETRICAL JOURNAL.

We have received from Dr. Clay, of Manchester, England, a prospectus of a journal, to be issued bi-monthly, devoted to Obstetric Medicine and Surgery, and the Diseases of Women and Children, and to be illustrated with wood cuts and engravings.

The enterprise is a new one in England as well as in the United States, although not without examples on the continent. The materials for such a record are and will continue to be abundant, and under the supervision of a competent editor, the journal must prove eminently useful.

The editor, Dr. Charles Clay, is extensively and advantageously known as a contributor to the British medical journals on various subjects of obstetrical science, and as we understand he is to have the assistance of many eminent medical men, British and Foreign, we have the best assurance that the work will be ably conducted. For the information of our readers, some of whom may be disposed to subscribe for the journal, we have inserted the "prospectus" in our Record. We shall welcome it as a valuable addition to our list of exchanges.

